# Addressing Infant and Young Child Feeding Recommendations From a Planetary Health Perspective

**DOI:** 10.1016/j.advnut.2024.100303

**Published:** 2024-09-13

**Authors:** Andrea Santos-Guzmán, Juan A Rivera, Mishel Unar-Munguía, Ivonne Ramírez-Silva

**Affiliations:** 1 Center for Health and Nutrition Research, National Instituto of Public Health, Cuernavaca, Morelos, Mexico; 2 Center for Research in Population Health, National Institute of Public Health, Cuernavaca, Morelos, Mexico

**Keywords:** environmental impact, breastfeeding, commercial milk formula, IYC feeding recommendations

## Abstract

Current international infant and young child (IYC) feeding recommendations consider nutrition and health but not environmental impacts. Only a handful of countries have dietary guidelines that provide quantitative recommendations for food groups of environmental concern. This study aimed to perform a narrative review of the environmental impacts of commercial milk formula compared with breastfeeding and to analyze the degree to which current country-specific IYC feeding recommendations are aligned with sustainable dietary targets. A mixed-methods review was conducted, including the following: *1*) a narrative review of the environmental impact of commercial milk formula compared with breastfeeding and *2*) a comparison of recommended intake of meats and dairy for children IYC based on country-specific dietary guidelines compared with the EAT–Lancet Commission dietary targets (ELCT) for children 24 mo or older and adults and if the ELCT should be adjusted for the energy needs of IYC. Formula feeding has a greater environmental impact (∼48% higher carbon footprint) than exclusive breastfeeding. Available country-specific dietary guidelines for meat and dairy products in children IYC are, in general, at or below the upper limits of the ELCT recommended ranges for older children and adults but are in most cases above the upper limit when adjusting for the energy needs of IYC. Exclusive breastfeeding should be protected and promoted not only as the healthier but also as the most sustainable alternative. Available complementary dietary feeding recommendations of nonprocessed meat and dairy fall below current ELCT for older children and adults. Given that IYC require a more nutrient-dense diet than older children and adults and considering the small quantities they consume, we recommend using the unadjusted ELCT as reference for IYC feeding while specific international consumption recommendations are developed for this age group.

**Statement of Significance**

The environmental impact of infant and young children’s feeding practices remains relatively unexplored and lies beyond the scope of the EAT–Lancet Commission Dietary Targets for planetary health. This review examines existing evidence on the environmental effects of early feeding, including the carbon footprint and other environmental impact indicators of exclusive breastfeeding compared with infant formula feeding, and evaluates whether current country-specific dietary guidelines for infant and young children, particularly regarding food groups of environmental concern, align with dietary targets for planetary health, and whether adjustments to these guidelines are necessary.

## Background

The EAT–Lancet Commission has established quantitative reference dietary targets for healthy and sustainable diets, which are composed largely of whole grains, fruits, vegetables, legumes, nuts, and unsaturated oils. It has also recommended a moderate intake of seafood and poultry and a reduction or elimination of red meat, processed meat, added sugar, refined grains, and starchy vegetables [1]. However, these reference targets apply to children 2 y and older. The commission adhered to global guidelines recommending exclusive breastfeeding (EBF) for the first 6 mo of life, continued breastfeeding ≤2 y of age or more [2,3], and for children aged 12–23 mo, followed the WHO complementary feeding guidelines for infants and young children (IYC), which propose the daily inclusion of ≥5 of the 8 food groups, including breastmilk (minimum dietary diversity) [4], without indicating portion sizes [3].

Breastfeeding recommendations are supported by strong evidence regarding health and development, as adequate breastfeeding practices are associated with reduced risk of overweight and obesity later in life, reduced risk of infections, and positive effects on cognition [[5], [6], [7]]. Mothers with longer periods of breastfeeding are also less likely to develop postpartum depression and hemorrhage, chronic diseases, and some types of cancers [6,8]. This practice also fosters societal development [9,10], as every $1 invested in protecting and promoting EBF is estimated to generate $35 in economic returns [11], and it can be considered a global food production system [12].

Despite extensive evidence supporting breastfeeding’s economic, social, and health benefits [5,[13], [14], [15]], some indicators of IYC feeding practices, such as EBF in the first 6 mo of age and continued breastfeeding at 2 y of age, still fall below global targets despite improvements in the last decade [16]. Since the mid-20th century, economic, demographic, and sociocultural factors have driven an increase in commercial milk formula (CMF) consumption [[17], [18], [19]]. Furthermore, the marketing strategies used by the CMF industry have significantly contributed to driving global consumption of formula. These practices include unsupported claims and solutions to common infant health challenges, use of digital platforms, engagement with health care providers, and other means to circumvent the International Code of Marketing of Breastmilk Substitutes (the Code), including interference with policies to regulate marketing of CMFs, which overshadows efforts to support, protect, and promote breastfeeding [20,21].

Because CMF is primarily produced from cow's milk, there are concerns about its environmental impacts. In the food system, dairy is the second largest greenhouse gas (GHG) emitter after meat, contributing to 3.1 gigatons CO_2_ equivalent/year (GtCO_2_eq/y), which translates into 3% of total global anthropogenic GHG emissions [22]. Early feeding also involves the introduction of complementary foods—including meats and dairy—for IYC starting at 6 mo old. Of the global GHG emissions stemming from food production (17,318 TgCO_2_eq/y), 57% are attributed to the production of animal-based food [23]. However, although breastfeeding is considered environmentally sustainable [24] and part of the triple-duty actions to address the global syndemic of obesity, undernutrition, and climate change [25], a gap in understanding the environmental impact of IYC feeding remains, as this aspect has not been thoroughly explored.

The WHO’s dietary guidelines for IYC, including the most recent edition [3], do not specify recommended amounts of food groups to meet the energy and nutrition needs of this age group. Given the variety of food sources, food availability, and accessibility across different regions and countries, developing such international recommendations poses a major challenge, especially if they seek to comply with dietary targets for planetary health. Although many countries have made efforts to modify and update their food-based guidelines based on the FAO’s process for developing dietary guidelines [26], it remains crucial to evaluate these from an environmental perspective [27].

Through a mixed-methods review, this article analyzes the environmental impact of CMF compared with that of breastfeeding and the degree to which the unadjusted and the IYC energy-adjusted EAT–Lancet Commission Dietary Targets (ELCT) for meats and dairy compare with country-specific recommendations for IYC.

## Methods

This is a mixed-methods review that includes a narrative review to investigate the environmental impact of milk formula feeding compared with breastfeeding. Results were obtained from quantitative studies on the environmental impact of production and/or consumption of CMF and breastmilk, globally.

Our inclusion criteria focused on original research studies and systematic or narrative reviews with quantitative data published in English that examined the environmental impact of breastfeeding (exclusive or partial) and CMF production and/or consumption. This included studies addressing various environmental impact indicators of breastfeeding compared with formula feeding. The key environmental impact indicators we were interested in were GHG emissions, carbon footprint (CFP), water (blue, green, and gray) footprint, and land use. However, our search strategy (Supplemental Figure 1) also included the terms novel entities, pollution, and waste to capture any additional environmental impact indicators. We excluded studies that focused on the environmental impact of complementary feeding products and dairy products other than CMF, as well as those evaluating pollutants in infant formula or breastmilk. The review was performed using scientific databases (PubMed, Science-Direct, Springer Link, and Scielo) from their origin to 26 July, 2024.

To assess whether current recommendations for complementary feeding of IYC are aligned with sustainable dietary targets, we evaluated whether the recommendations fall outside the ranges of intakes proposed by the ELCT for older children and adults. We analyzed country-specific dietary guidelines that provided nutritional recommendations in daily or weekly amounts for IYC aged 6–24 mo. To identify these guidelines, we conducted a search in the FAO’s food-based dietary guidelines repository and downloaded the available documents [28].

Initially, we identified countries with either separate dietary guidelines for IYC or dietary recommendations for this age group within general population guidelines (*n* = 46). From these, we selected guidelines that included quantitative dietary recommendations (*n* = 9) and further refined our selection by excluding those where recommendations were presented as composed meals or interchangeable lists (*n* = 3) and retaining those that provided specific portion sizes or recommendations in grams per day or per week by food group (*n* = 6) (Supplemental Table 1 for further detail). In the initial search, we were unable to access 18 links from the 96 countries with guidelines. During a subsequent review, we accessed 9 of these links, 2 of which included information on portion sizes by food group and were thus included in our analysis. We reviewed guidelines available in English, Spanish, Italian, French, German, and Portuguese. However, we did not review guidelines in other languages with different alphabets that did not have a version in any of these languages.

The countries included in the analysis were India [29], Panama [63], the United States [30], Paraguay [31], Zambia [32], and Mexico. The most recent IYC dietary guidelines from Mexico were obtained from the research team responsible for the development of the 2023 Healthy and Sustainable Dietary Guidelines for the Mexican population, which have already been published [33]. However, the specific guidelines tailored for IYC have not yet been released.

We analyzed the recommended amounts in grams per week for the following food groups: *1*) beef and lamb, *2*) pork, *3*) chicken and other poultry, *4*) eggs, *5*) fish, and *6*) milk. Initially, we compared them with the unadjusted ELCT values for a 2500-kcal diet based on planetary health, both for the average value and the upper limit of consumption. We also compared country-specific recommendations with the ELCT amounts after adjusting for estimates of energy recommendations for IYC, as follows: 6–8 mo, 641 kcal; 9–11 mo, 711 kcal; and 12–24 mo, 868 kcal [34].

## Results

### Environmental impact of early life feeding modes

A total of 4195 scientific articles were identified in the initial search. After screening titles and abstracts, 14 articles were selected for full-text review. Ultimately, 9 articles that met the eligibility criteria were selected (Supplemental Figure 1). Four articles examined the environmental impact of exclusive infant formula consumption and production compared with that of EBF [[35], [36], [37]]. Two articles assessed the environmental impact of infant, special, and follow-up milk formula only [38,39], 1 article assessed the CFP and water footprint of infant formula [40], 1 scoping review analyzed the environmental impact of dairy and CMF [41], and 1 state-of-the-art review described the implications of not breastfeeding on the environment [42] (Table 1) [[35], [36], [37], [38], [39], [40], [41], [42], [43]].

**TABLE 1:** Summary of findings of the systematic literature review of studies analyzing the environmental impact of commercial milk formula and breastfeeding.

Author	Journal	Publication year & aim	Country	Methods	Comparators	Environmental outcomes evaluated	Results	Limitations	Conclusions
Long et al. [43]	*Journal of Cleaner Production*	2021 To consider the previously unstudied moral implications of an engineering intervention to improve production from an environmental perspective	Ireland and China	Comparison of emissions saved by decarbonizing BMS industrial processing with renewable gas to those saved by increasing exclusive breastfeeding to 50%. Framework was based on existing carbon accounting in energy models. BMS consumption per child was estimated using IPCC nomenclature, considering variables such as annual BMS consumption, the number of infants, exclusive breastfeeding rates, and monthly BMS consumption per infant. It also considered fuel consumption in BMS production and emissions from BMS consumption per country	Decarbonizing current consumption of BMS produced in Ireland with renewable gas Achieving 50% exclusive breastfeeding rate Achieving 50% EBF rate and displacing natural gas in the remaining product	GHG^3^ emissions saved (energy only) GHG emissions saved—energy and agriculture (including increased diet of the mother)	Emissions saved by reaching the minimum global breastfeeding target (1444 t CO_2_) outweigh those save by using renewable gas to replace natural gas in the production of BMS (918.9 tCO_2_) in Ireland.	This study considered only the first 6 mo of infant feeding, so future work could explore potential emissions savings from decarbonizing the follow-on and toddler milk	BF support can be considered a demand-side measure for mitigating climate change by reducing the demand for energy services to produce BMS
Karlsson et al. [36]	*Journal of Cleaner Production*	2019 To estimate the GHG emissions, or CFP, from production and consumption of BMS.	New Zealand, United States, Brazil, and France (CFP of BMS production) United Kingdom, China, Brazil, and Vietnam (CFP of BMS consumption)	LCA methodology assessing environmental impact of BMS, including the use of natural resources, energy, land, minerals, metals, products, emissions, and waste. The study was performed and documented according to ISO 14067 standards, with impacts aggregated into different environmental categories related to 1 kg of formula. Global warming potential was assessed over a 100-y timeframe using IPCC AR4 factors, and results were tested for sensitivity. The study was developed in 3 stages: partial CFP from producing and packaging 1 kg BMS; CFP from consuming 1 kg BMS, which includes emissions from productions, transport to retail, infant bottle production/sterilization, and heating water; and CFP from breastfeeding to replace 1 kg BMS.	Breastfeeding BMS	The CFP of production (raw milk, vegetable oils, processing, packaging, and transport). CFP of consumption (production, sterilization, preparation, transport, and bottle production). CFP from breastfeeding (CFP^5^ of additional food recommended for breastfeeding mothers to replace 1 kg of BMS)	Production emissions (CO_2_ per kg of BMS) varied from 7.0 ± 1.0 in the United States to 11 ± 2.0 in Brazil. Consumption emissions also varied from 11 ± 1.0 in the United Kingdom and Vietnam to 14 ± 1 in Brazil and China. Breastfeeding emitted less CO_2_ equivalent per kilogram of BMS compared with formula feeding, from 5.9 ± 1 in Vietnam to 7.8 ± 1.5 in Brazil. Feeding an infant with BMS for 6 mo generated 226–288 kg CO_2_ eq, whereas exclusive breastfeeding for the same duration resulted in 123–162 CO_2_ eq/kg. The net benefit of exclusive breastfeeding ranged from 95 to 153 CO_2_ eq/kg.	The study only assessed the climate impact of different infant feeding approaches, but it did not consider pollution and contamination of waterways and soils, biodiversity loss and use of limited or nonrenewable resources such as land, water, and fossil fuels.	Breastfeeding has a consistently lower CFP than using BMS. It is advisable to consider that food production also affects the environment in many other ways, so it is recommended to do a full comparison of the environmental impact of BMS.
Dadhich et al. [38]	*Journal of Human Lactation*	2021 To describe exploratory estimates of GHG emission factors for all infant and young child milk formula products and estimate national GHG emission association with commercial milk formulas sold in selected countries in the Asia Pacific region	Australia, South Korea, China, Malaysia, India, and Philippines	Secondary data analysis to estimate CO_2_ emissions per KF of milk formula, using LCA methodology. GHG emission factors for milk powder, vegetable oils, and sugars were determined via literature review. Ingredient proportions were calculated according to FAO Codex Alimentarius guidance. Analysis proceeded in 4 stages: identifying milk formula composition, calculating ingredient contributions, identifying emission factors, and estimating annual emissions based on sales date from 2012 to 2017 for 6 selected countries.	Total annual GHG emission (kg CO_2_ eq) of standard milk formula/special infant formula and follow-up formula (for each country) in 2012. Projected annual GHG emissions of standard milk formulas/special infant formula and follow-up formula (for each country) in 2017.	Annual GHG emissions (years 2012 and 2017) by country	In 2012, total emissions amounted to 2,893,030 kg CO_2_ eq, with China being the largest emitter. By 2017, forecasted emissions surged to 4,968,534 kg CO_2_ eq, with China leading in emissions. The projected increase in milk formula emissions from 2012 to 2017 is substantial, with a 72% rise in total emissions. Special formula emissions are expected to increase by 53%, whereas follow-on formula emissions are projected to surge by 78%.	Results hinge on the validity of the Euromonitor data. It also excluded nonretail supply of milk formula (like donations to health facilities), which means considerable underestimation of GHG emissions. Estimates are partial and exclude postmanufacturing phases of the milk formula product life cycle.	Policies, programs, and investments to shift infant and young child diets toward less manufactured milk formula and more breastfeeding are triple-duty actions that help improve dietary quality and population health and improve the sustainability of the global food system.
Andresen et al. [35]	*International Journal of Environmental Research and Public Health*	2022 To compare the environmental impact of 4 mo of infant formula feeding with 4 mo of exclusive breastfeeding.	Norway	LCA methodology to evaluate environmental impact across 5 functional units: 1 kg powdered infant formula at retail, 1 kg ready-to-consume infant formula, 1 kg breastmilk, 4 mo of exclusive formula feeding, and 4 mo of exclusive breastfeeding in Norway. The analysis comprised 4 steps: assessing the environmental impact of (1) powdered infant formula production and distribution, (2) preparing ready-to-consume formula (including bottle preparation), (3) breastfeeding, and aggregating results to determine the total impact over 4 mo.	Consumption of 1 kg of infant formula vs. 1 kg of breastmilk Exclusive breastfeeding (4 mo) vs. exclusive infant formula feeding (4 mo)	Key environmental indicators between the 2 feeding practices. For both 1 kg of infant formula and 1 kg of breastmilk, the global warming potential was measured in kg CO_2_ eq, marine eutrophication in kg P-equivalent, and land use in m2a crop-equivalent. Additionally, the study contrasts the impacts of exclusive infant formula feeding with exclusive breastfeeding using the same environmental metrics.	Infant formula had a higher environmental impact than breastmilk. For 1 kg, infant formula’s global warming potential was 2.02 kg CO_2_ eq vs. breastmilk’s 1.58 kg CO_2_ eq, with higher impacts also seen in terrestrial acidification, freshwater and marine eutrophication, and land use. Exclusive formula feeding resulted in 200 kg CO_2_ eq global warming potential compared with 145 kg CO_2_ eq for breastfeeding, along with greater impacts in all other measured categories.	Use of secondary LCA data for raw ingredients and processes.	Breastfeeding has a lower environmental impact than feeding with infant formula.
Cadwell et al. [39]	Breastfeeding medicine	2020 To compute a minimal estimate of GHG emissions for powdered infant formula products sold in North America comprising Canada, Mexico, and the United States	Canada, Mexico, and the United States	Industry data from Euromonitor International of powder formula sold in 2016 in North America was consulted to gather information on powdered formula sales in the regions. Recipes for powdered formula blends were collected based on these data. A literature review was then conducted to assess the emission contribution of each ingredient according to its percentage inclusion. Carbon emissions resulting from the production of each powdered formula blend were calculated. Based on sales figures, the CFP of each type of powdered formula in North America was computed. Finally, measurements of CO_2_ were translated using a GHG equivalencies calculator for comprehensive assessment.	Four types of formula milk: Standard powder; follow-on powder; growing-up powder; and special powdered total emissions	Estimated of GHG emissions for standard powder milk, follow-on powder milk, growing-up powder milk, and special powder milk, all measured in CO_2_ equivalents per kilogram. The total GHG emissions for these types of powdered milk were also calculated.	For standard powder milk, the United States led with 412,614 CO_2_ eq/kg. In the follow-on powder milk category, Mexico was the highest emitter with 99,424 CO_2_ eq/kg. Mexico also topped the growing-up powder milk emissions at 239,844 CO_2_ eq/kg. For special powder milk, the United States had the highest emissions at 201,770 CO_2_ eq/kg. Overall, the United States contributed the most to total emissions with 655,956 CO_2_ eq/kg, followed by Mexico at 435,829 CO_2_ eq/kg, making them the primary contributors to the region’s total of 1,161,932 CO_2_ eq/kg.	Only the common types of powdered formula were considered. The emissions data for formula ingredients were limited to that which was publicly available and some of the data used may be dependent on local production and manufacturing techniques. Calculations were based on the amount of powdered formula sold at retail, not wholesale sales or free supplies to health facilities. No comparison of CO_2_ emissions to breastfeeding was made.	Environmental and GHG impact of powdered infant formula, and related hazards arising from climate change, can be a relevant factor for health care providers in their advice to families on infant feeding. The results of this study should be considered when developing and funding infant and young child feeding policies and supportive programs.
Pope et al. [41]	*International Journal of Environmental Research and Public Health*	2021 To examine the impact of commercial milk formula use on planetary health, considering in particular its effects on climate change, water use, and pollution and the consequences of these effects for human health.		Search of the Medline and Embase databases from their origin to May 2021, conducted for peer-reviewed literature that investigated the environmental impact of commercial milk formula and dairy industry.	Commercial milk formula (CMF) and breastmilk feeding	CMF-GHG emissions CMF-water use CMF-other environmental impacts Proposed mitigation strategies	Consuming 1 kg of CMF (cow milk) emits 11–14 CO_2_ eq/kg. Added vitamins and minerals contribute to 1.3 kg CO_2_ e/kg CMF. The water footprint for 1 kg of CMF use is 699 L. CMF production emits air pollutants, including particulate matter, NOx, and volatile organic compounds, with potential health impacts. The review emphasizes the need to reduce CMF demand and promote sustainable alternatives, such as breastfeeding.		Facilitation and protection of breastfeeding represent a key part of developing sustainable first-food systems and have potential benefits for maternal and child health. Reducing demand for dairy is deemed essential to stay within planetary boundaries. Advocating for that transition in food systems is a main role of global public health.
Amonkar et al. [37]	*Journal of Environmental Accounting and Management*	2019 To use GHG emissions as a comprehensive climate change relevant metric for comparing the 2 methods (breastfeeding and formula feeding) referred to as “nursing methods” to guide government officials and nongovernmental organizations to make more informed policy and outreach decisions.	United States	LCA methodology to compare the cradle-to-grave GHG emissions impacts of breastmilk production, pumping, refrigeration/reheating, and bottle feeding against formula milk production, preparation, and bottle feeding. The functional unit for comparison was the average milk feeding amount of a 2–6-mo-old infant per day (30 oz). The analysis considered 5 categories and 8 parameters: mother, infant, milk, breast pump, and infant bottles. GHG emissions were analyzed across 8 life cycle stages, including packaging materials, formula ingredients, transportation, manufacturing, retailing, use phase, and end of life. Emissions from each stage were aggregated to determine cradle-to-gate GHG emissions per functional unit, using a combination of primary and secondary data.	Three scenarios (g CO_2_eq/d based on parameters that represent different practices) for both nursing methods. 1) Low scenario 2) Base case 3) High scenario	Low scenario (g CO_2_e/d) Base case scenario (g CO_2_e/d) High scenario (g CO_2_e/d)	Breastmilk: 1463 Formula milk: 873 Breastmilk: 1858 Formula milk: 1136 Breastmilk: 2202 Formula milk: 1400	The analysis was not conducted for the possible variation in distances between the grocery store and mother’s home. People from different states or cities can have different CFPs, and different shopping habits. This could also impact the total GHG emissions.	Breastmilk feeding is more GHG intensive than formula milk feeding, except for those mothers who can manage without any pumping/storing. Even at the maximum potential GHG emissions from formula milk feeding, the impact was still less than the minimum potential impact of breastmilk feeding. However, breastmilk nursing has significant health benefits over formula milk feeding, so the results should not be used to say that one is better than the other.
Smith et al. [40]	*Frontiers in Nutrition*	2024 To provide an overview of the Global Footprint Tool (GFT) development, key features, and potential uses.	LMIC	DMADV methodology: define, measure, analyze, design and verify to develop the GFT to calculate the CFP and water footprints of CMF for infants under 6 mo at national levels to support breastfeeding policies. The tool’s calculations are based on current LCA of CMF production and its significant water and carbon demands.	CMF for infants under 6 mo	CFP and water footprint	The GFT showed that CMF use generates 6–8 billion kg of CO_2_ and consumes over 2562 billion L of water annually. The GFT provides tools to assess the environmental impacts of policies and interventions affecting infant feeding, as well as a global scenario where formula use displaces all BF in LMICs, potentially increasing emissions to 29.37 billion kg of CO_2_ and water use to 12.5 billion L annually.	The GFT relies on open-source data, lacking coverage for high-income countries, and does not account for emissions from follow-on formulas or growing-up milk. It overlooks the environmental impact of child spacing from increased breastfeeding and excludes the GHG effects of expressing and donating human milk.	The GFT highlights the benefits of interventions that support BF, such as paid maternity leave and peer counseling, in reducing the demand for carbon-intensive and water-scarce CMF products.
Bai and Alsaidi [42]	*Journal of Human Lactation*	2023 To describe the implication of not breastfeeding on the environment within the context of food system sustainability.		State-of-the-art review of studies examining the environmental impact of artificial milk formula vs. breastfeeding.	Breastfeeding vs. BMS feeding	Breastfeeding and CFP reduction Breastfeeding and water usage reduction Breastfeeding and natural birth interval	Breastfeeding reduces the CFP compared to artificial milk formulas, which require substantial land, water, and generate high GHG emissions. Breastfeeding for 6 mo can significantly reduce CO_2_ emissions and water usage. Mass production of artificial milk formula exacerbates water pollution and has a significant water footprint, requiring ∼800 L of water to produce 1 L of cow milk and 4700 L/kg of milk powder. Breastfeeding can save ≤105,280 L of water over 6 mo. Breastfeeding duration is associated with longer birth intervals, reducing population growth and conserving resources.	The recent emergence of the concept of environmental cost of not breastfeeding resulted in few studies on the topic. Most articles focused on health, social, and economic benefits of breastfeeding rather than environmental impact.	Substituting artificial milk formula for breastfeeding increases CFP and waterway degradation. Promoting exclusive breastfeeding for the first 6 mo can sustain the global food system.

Abbreviations: BMS, breastmilk substitute; CFP, carbon footprint; CMF, commercial milk formula; GHG, greenhouse gas; IPCC, Intergovernmental Panel on Climate Change; LCA, life cycle assessment; LMIC, low-income and middle-income country.

The CFP of infant formula is mainly due to raw milk production, but it also includes vegetable oil production, dairy processing, transport, formula processing and packaging, transportation to the point of sale, sterilization, preparation, and bottle production [36]. Breastfeeding requires an additional 500 kcal/d for the lactating mother to produce milk [35]. This extra energy intake contributes to the CFP attributable to breastfeeding.

Using life cycle assessment (LCA) methodology, a study demonstrated that the CFP of exclusive infant formula consumption ranged between 11 and 14 kg CO_2_eq/kg of prepared formula, whereas EBF’s CFP was between 5.9 and 6.9 CO_2_eq/kg of breastmilk [36]. Total blue water footprint for 1 kg of formula use, from raw milk production to bottle washing, was equivalent to 699 L of water extracted from surface or groundwater sources [41].

By 2012, emissions attributable to CMF sold in 6 Asia Pacific countries were equivalent to emissions from ∼6.9 billion miles driven in car, which are generated mainly in the production phase [38]. Another study showed that the CFP associated with exclusive formula feeding and EBF for 6 mo ranged between 226 and 288 CO_2_eq and 123 and162 CO_2_eq per infant, respectively, which implies a net difference of 95–153 CO_2_eq (∼48%) [36].

In 2016, in North America, the annual CO_2_ emissions attributable to CMF consumption sold at retail totaled 1,161,936 tons of CO_2_eq (59.06 kg CO_2_eq per capita). Emissions from the consumption of growing-up milk (for children aged older than 12 mo) were much higher in Mexico, whereas emissions from the consumption of infant formula (0–12 mo) were higher in the United States and Canada [39]. According to a study carried out in Ireland, achieving the minimum global EBF target for 2025 (50% of children under 6 mo) projects further savings in CO_2_ emissions (∼58%), relative to optimizing formula production with renewable gas [43].

The Green Feeding Climate Action Tool (GFT) is a novel tool that complements 3 existing nutrition tools, which quantify health and economic losses due to not breastfeeding but do not address environmental impacts. The GFT fills this gap by calculating CFPs and water footprint from CMF. After development, the GFT was validated by comparing country estimates from published studies to understand sources of variance and standardize estimates. Although feedback continues to be collected for ongoing improvement, the GFT has already yielded important results [40].

Specifically, the GFT assessed the environmental impact of infant formula use among infants younger than 6 mo. For 80 countries, mainly low-income and middle-income countries, infant formula use resulted in annual emissions of 5925–7541 million kg CO_2_eq and water consumption of 2562.5 billion L. The tool also allows for the input of local breastfeeding data for more accurate estimations. In China, improving EBF rates to 90% could potentially reduce annual GHG emissions from 437.4–556.7 million kg to 118–150.7 million CO_2_eq and water use from 189,000 million L to 51,167 million L. Policy changes implemented in Canada in 2008, which increased EBF from 23.1% to 31.5%, could have reduced Canada’s annual GHG emissions by 3.9–4.0 million kg CO_2_eq and water use by 1661.7 million L [40].

Only 1 of the 7 reviewed studies concluded that EBF has higher GHG emissions (1858 CO_2_eq/d/infant) than exclusive formula feeding (1136 CO_2_eq/d/infant), even when accounting for the maximum potential GHG emissions from the latter. This study considered the use of electric pumps and refrigeration for extracted breastmilk [37]. However, results from this analysis are based on a United States setting, where there is no statutory paid parental leave, only one-sixth of employed women have access to paid leave [44], and 85% of breastfeeding mothers pump, store, and reheat breastmilk.

Other environmental impact indicators, such as terrestrial acidification, freshwater and marine eutrophication, water footprint, and land use have been estimated by comparing exclusive formula feeding with those of EBF. Results showed that the environmental impact of 4 mo of formula feeding for the listed indicators was 35%–72% higher than that of EBF for the same period, with the largest difference seen in terrestrial acidification [35]. Furthermore, a review on the impact of not breastfeeding also examined the role of breastfeeding in birth spacing. It established that longer breastfeeding duration is associated with longer birth intervals, which reduces populations growth and conserves resources [42].

### Complementary feeding for children 6 to 24 mo: recommended intake of meat and dairy

Energy requirements for breastfed children older than 6 mo that must be covered by complementary foods range from 200 to 550 kcal/d, depending on the children’s age. IYC should be fed 2–4 times/d, with additional nutritious snacks [45], as desired. The most recent guidelines recommend starting with pureed, mashed, and semisolid foods and gradually transitioning to finger foods and foods consumed by the rest of the family, while continuing to breastfeed [3]. The latest WHO recommendations suggest that meat, poultry, fish, or eggs should be eaten daily or as often as possible, as well as vitamin A–rich fruits and vegetables. Legumes are a good source of protein and dietary fiber, and sources of long-chain-polyunsaturated fatty acids should be consumed to promote cognitive and motor development. Dairy foods (yogurt and cheese) can be introduced before 12 mo, and follow-up formulas (formulas aimed at children 12–23 mo) are not recommended [2,3].

### Comparative analysis of the country-specific dietary guidelines recommendations and the unadjusted ELCT values

Table 2 presents recommendations for meats and dairy for children aged 6–24 mo from dietary guidelines in Panama (6–24 mo), the United States (12–23 mo), India (6–36 mo), Paraguay (12–24 mo), Zambia (6–23 mo), and Mexico (6–24 mo), alongside unadjusted and energy-adjusted ELCT recommendations. The dietary recommendations for IYC vary between countries. In the United States, these guidelines specifically apply to children who are no longer receiving breastmilk or infant formula. In India, the recommendations are for children who are not being breastfed. In Zambia, Panama, and Mexico, guidelines for children aged 6–8 and 9–11 mo consider those still breastfeeding, whereas those for 12–24 mo focus on children who are no longer breastfed. Paraguay provides recommendations for children who are being breastfed.

**TABLE 2:** Comparison of energy-adjusted and unadjusted ELCT recommendations with country-specific dietary guidelines for meat and dairy intake in infants and young children.

Dietary guidelines	Age group	Total caloric intake (kcal/d)		Meats (g/wk)					Dairy (mL/wk)
				Beef and lamb	Pork	Chicken and other poultry	Eggs	Fish	Whole milk or derivative equivalents
Dietary Guidelines for children under 2 y (Panama)	6–8 mo	403		56	—	112	126	56	BM
	9–11 mo	543		112	—	224	126	112	BM
	12–24 mo	760		56	—	112	126	56	1750
American Dietary Guidelines (United States)	12–23 mo	1,000		217^1^			70	84	1750
				108.5^2^		108.5^2^			
Dietary Guidelines for Indians	6–12 mo	676		87.5^3^					2,800
				21.9^2^		21.9^2^	21.9^2^	21.9^2^	
	1–3 y	1,060		350^3^					3,500
				87.5^2^		87.5^2^	87.5^2^	87.5^2^	
The Mexican Healthy and Sustainable Dietary Guidelines	6–8 mo	625		15	15	45	30	18	125
	9–11 mo	708		30	30	60	60	35	250
	12–24 mo	930		30	40	60	120	35	3860
Zambia Food-based Dietary Guidelines	6–8 mo	600		161^4^					0
	9–12 mo	700		161^4^					427
				40.3^2^		40.3^2^	40.3^2^	40.3^2^	
	13–23 mo	900		322^4^					1,708
				80.5^2^		80.5^2^	80.5^2^	80.5^2^	
Dietary Guidelines of Paraguay	12–24 mo			250^5^			150		4,200
				83.3^2^		83.3^2^		83.3^2^	
ELCT	Adults ≥18 y	2500	Average	49	49	203	91	196	1750
			Upper	98	98	406	175	700	3500
	**Energy intake recommendations**								
Energy-Adjusted ELCT	6–8 mo	641	Average	12.5	12.5	52	23.3	50.3	448.7
			Upper	25.1	25.5	104	44.9	179	897
	9–11 mo	711	Average	13.9	13.9	57.7	25.9	55.7	497
			Upper	27.9	27.9	115.5	50	199	995
	12–24 mo	868	Average	17.0	17.0	70.5	31.6	68.1	607.6
			Upper	34	34	141	61	243	1,250

Abbreviations: ELCT, EAT–Lancet Commission Dietary Targets for Planetary Health.

1 Includes poultry and meats.

2 Quantities represent the combined values of meats, assuming the grams were evenly distributed among the products included for each country.

3 Includes eggs, meat, chicken, or fish.

4 Includes meats, fish, and eggs.

5 Includes meats, fish, and chicken.

In Panama, meat recommendations for children aged 6–8 and 12–24 mo exceeded average unadjusted ELCT values but remained below the upper limit. For infants aged 9–11 mo, recommendations surpassed the upper limit by 14%. In the United States, meat and poultry recommendations for children aged 12–24 mo are combined. These recommendations remained below the unadjusted ELCT upper limit for the sum of meats and poultry (301 g/wk) and below the average for eggs and fish.

India combines all animal-source foods for children aged 6–11 mo and 1–3 y, consistently staying below unadjusted ELCT average recommendations. In Mexico, recommendations for animal-source foods across age categories consistently remained below the unadjusted ELCT average, except for the recommendation for eggs for children aged 9–24 mo, which still fell within the ELCT upper limit. In Zambia, the meats included are beef, fish, and eggs. For children aged 6–12 mo, the total animal-source food recommendation was below the average of unadjusted ELCT values. For children aged 1–2 y, both the total and even distribution of animal-source foods recommendations remained below the average and upper limits of the unadjusted ELCT values. In Paraguay, the recommended meats include beef, fish, and chicken. For children aged 1–2 y, both the total and even distribution of recommendations remained below the average and upper limits of the unadjusted ELCT values.

Milk recommendations across most countries and age groups did not exceed the unadjusted upper ELCT value. In India, dairy recommendations for 1–3-y-old children aligned with the upper ELCT recommendation; those for 6–12-mo-old infants exceeded the average value but stayed within the upper ELCT limit. In Mexico, dairy recommendations for infants aged 1–2 y exceeded both the average and upper unadjusted ELCT recommendations, but only by 10%. In Zambia, there are no dairy recommendations for children aged 6–8 mo. For children aged 9–12 and 13–24 mo, the dairy recommendations remained below the average and upper limit of the unadjusted ELCT values. In Paraguay, the dairy recommendation for children 1–2 y exceeded the upper unadjusted ELCT value by 20%.

### Comparative analysis of the country-specific dietary guidelines: recommendations and the adjusted ELCT values

The ELCT values adjusted for estimated energy requirements (referred to hereafter as adjusted ELCT values) consistently indicate lower quantities than both the nonadjusted ELCT values and country-specific recommendations across all food groups. Specifically, the adjusted ELCT values for meat, eggs, and milk are notably below the recommendations outlined in the dietary guidelines for each country and age group. Throughout, the recommendations set by each country surpassed both the average and upper adjusted ELCT values, except for the upper limit for fish.

In Mexico, the recommendations for chicken and poultry closely approached or fell below the adjusted ELCT recommendations. Similarly, in Zambia, the recommendations for fish and chicken remained below or near the adjusted upper limit ELCT values for children aged 6–8 and 9–12 mo. However, for all age groups and countries, the recommendations for dairy generally exceeded the adjusted ELCT values, except for children aged 6–8 and 9–11 mo in Panama, Mexico, and Zambia.

## Discussion

This review indicates that exclusive infant formula feeding has a greater environmental impact and higher blue water footprint than EBF. This holds even when considering the CFP associated with the increased energy requirement by breastfeeding mothers for milk production.

The CFP of EBF is only higher when considering the use of electric tools and equipment involved in expressing and storing human milk. This practice is common among working mothers for whom it is not possible to engage in direct breastfeeding. However, despite the potential greater environmental impact of pumping and storing breastmilk, it is crucial not to overlook the significant health gains that breastfeeding offers to both mother and infant, ones that cannot be replicated by infant formula [20]. This finding emphasizes the environmental importance of implementing public policies that foster supportive environments for those aiming to breastfeed, such as paid maternity and paternity leave.

In the United States, the availability of paid maternity leave has shown associations with improved rates of breastfeeding initiation and duration [44] as well as the mental and physical health of both mothers and children [44,46,47]. Systematic reviews in low- and middle-income countries have found that extending maternity paid leave improves breastfeeding practices [48,49]. Moreover, the GFT analysis showed that in Canada, extended paid maternity leave resulted in a reduction of CO_2_ emissions and a substantial reduction in water consumption annually [40].

To lower the GHG emissions associated with CMF, including special formula, follow-up, and growing-up milk, it is essential to establish regulations that restrict the use of misleading nutrition and health claims in formula marketing [21,50,51]. Such regulations are critical in preventing the overprescription and overconsumption of these products [52,53], as their effectiveness in providing young children with nutrients is no greater than that of natural foods, making their consumption unnecessary [54].

Studies suggest that additional evidence is necessary to determine other environmental consequences caused by formula production and consumption, such as pollution and contamination of waterways and soils, loss of biodiversity, use of limited resources, and packaging waste [36]. According to the International Baby Foods Action Network (IBFAN), in 2008, 550 million infant formula cans, 86,000 tons of metal, and 364,000 kg of paper from formula packaging were added to landfills every year [55]. However, due to increased demand for CMF this estimate could be significantly higher at present.

Complementary feeding practices are also a crucial stage for young children. Meeting the energy demands during this stage is vital while ensuring optimal nutrient intake and minimizing impacts on the natural Earth systems. The findings of the comparative analysis between ELCT and country-specific dietary guidelines for meat and dairy recommendations indicate that if the unadjusted ELCT for older children and adults are adopted for IYC, current country-specific dietary guidelines for this age group fall within the ELCT upper limits, with exceptions for meat values in one age subgroup and dairy values in two age subgroups. Furthermore, the countries assessed used robust methodologies to develop their guidelines, which take into account not only specific nutritional recommendations for IYC but also factors such as food bioavailability and cultural dietary practices, including breastfeeding.

The ELCT consider an energy intake of 2500 kcal, whereas IYC have much lower energy requirements. Therefore, we also used the adjusted ELCT values to match the caloric needs of children aged 6–24 mo as a target. When these adjusted targets were used, they consistently fell below the current country-specific recommendations for meat and dairy. If adjusted ELCT values were applied, it could restrict adequate nutrient intake for IYC. Moreover, the idea of adjusted ELCT values overlooks the fact that IYC need a more nutrient-dense diet than older children or nonpregnant, nonlactating adults. This is due to their high nutrient requirements and small food intake, particularly for certain challenging micronutrients.

Animal-source foods, such as meat for iron and zinc, and dairy for calcium (for nonbreastfed IYC), are the best sources for these nutrients. Therefore, our advice is to consider the current ELCT values for older children and adults as possible upper limits for IYC when developing country-specific dietary guidelines for key food groups that may have an impact on the environment. It would also be beneficial to develop specific global targets for the complementary feeding period that consider both breastfeeding and complementary foods. This is particularly relevant because of the substantial difference between a 2500-kcal requirement for adults and a 600- to 1000-kcal intake for IYC. This approach is preferred rather than restricting recommendations to energy-adjusted ELCT values.

Additionally, according to the United Nations [56], children younger than 2 y comprise 3.3% of the world’s population, resulting in a relatively low environmental impact of their diet. Therefore, even if the ELCT for older children and adults are adopted for IYC, the environmental impact would be very small. Thus, there is no need to restrict meat and dairy products in this age group to reduce environmental impact. However, processed meats should be excluded from IYC's diet, as they have been classified as a carcinogen for humans by International Agency for Research in Cancer (IARC) of WHO [57].

It is important to note that consumption of meats and dairy among IYC are limited in settings with food insecurity, humanitarian crises, or populations where plant-based diets are predominant [2]. To prevent and address nutritional deficiencies in these contexts, international guidelines recommend the administration of nutrient supplements, small-quantity lipid-based nutrient supplements or foods fortified with vitamins and minerals containing iron, micronutrient powders, or lipid-based nutrient supplements to improve the quality of foods available [2,3,45,58,59]. Recommendations that limit the intake of meats and other animal-source foods should pay attention to micronutrient dietary intakes and evaluate the need to provide micronutrient supplements or fortified foods [60]. The WHO proposes specific recommendations for vitamin A, iron, iodine, and zinc during pregnancy and for children younger than 2 y [61,62].

### Limitations

This study has several limitations. First, there are few studies comparing the environmental impact of EBF with exclusive formula feeding and none that address mixed or continued breastfeeding, limiting our understanding of broader environmental implications. These studies provide us with upper limit estimates of the environmental impact for exclusive formula feeding. However, we included studies that examined the environmental impact associated to follow-up formula and growing-up milks, providing insights into the environmental impacts of continued formula consumption at older ages.

Second, in our analysis of IYC feeding recommendations compared with ELCT targets, a major limitation was the absence of global guidelines on specific food quantities for high-environmental impact foods (meats and dairy) from WHO and other UN agencies, which required us to rely on country-specific dietary guidelines. As a result, our finding may not fully capture the diversity of countries and food cultures, largely due to the absence of quantitative IYC feeding practice guidelines in many countries. Finally, variations in age and food group classifications across different guidelines further restricted comparability. The sustainable dietary targets we used (ELCT) were developed for children older than 2 y and adults, and there are no specific complementary feeding recommendations for various infant feeding categories (breastfeeding, partial breastfeeding, formula feeding, and nonformula dairy), each of which provides a wide range of energy and nutrients.

Despite these limitations, we consider that the guidelines developed by countries are based on thorough analyses of the energy, macronutrient, and micronutrient requirements of IYC. Therefore, this comparison can offer valuable insights for developing global dietary guidelines focused on planetary health. Moreover, we see our analysis as a step forward in providing information on the environmental benefits of EBF and as a starting point for addressing the specific needs for complementary feeding while considering the impacts of the Earth’s natural systems.

### Conclusions and recommendations

Early stages of life play a vital role in the growth and development of IYC. To maximize their potential and ensure optimal growth, it is crucial to meet their nutritional requirements in both quantity and quality. EBF should always be the primary choice for initial feeding, whenever possible. In specific cases, alternative options can be recommended by unbiased health care professionals, free from commercial interests. Encouraging exclusive and continued breastfeeding not only safeguards the health of infants and their families but also benefits the environment by minimizing GHG emissions associated with unnecessary consumption.

Nutritional recommendations should be approached with a focus on health, equity, and environmental protection. It is important to note that IYC should not be restricted from consuming animal-source foods, as their consumption does not pose a threat to planetary boundaries.

## Author contributions

The author's responsibilities were as follows: JR conceived the aim and scope of the article. JR, ASG, and MUM developed the methodology and design, with ASG drafting the initial manuscript. IRS reviewed the manuscript and refined the table containing the dietary guidelines values. All authors contributed to finalizing the manuscript's content, and they read and approved the final version.

## Funding

This research is funded by Bloomberg Philanthropies (Grant Number 71206). The funders had no role in study design, data collection and analysis, decision to publish, or preparation of the manuscript.

## Conflict of interest

The authors report no conflicts of interest.
