# Supplementary material for: Addressing Infant and Young Child Feeding Recommendations From a Planetary Health Perspective
Source: Adv Nutr. 2024 Sep 13;15(11):100303. doi: 10.1016/j.advnut.2024.100303 (PMC11656346; doi:10.1016/j.advnut.2024.100303)
Supplement: multimedia component 2 [file mmc2.docx]

**Supplementary Material 1**

The following data bases were used to obtain studies on the environmental impact of early feeding modes: PubMed, Science-Direct, Springer Link, and Scielo. The search strategy was carried from their origin to July 2024 as follows:

((infant formula) OR (milk formula) OR (breastmilk)) AND ((carbon footprint) OR (life cycle assessment) OR (pollution) OR (environmental impact) OR (climate change) OR (greenhouse emissions) OR (novel entities boundary) OR (planetary systems) OR (waste))

**Figure 1.** Flow Diagram of the systematic search of environmental impact of early feeding modes

**Results from systematic search**

(n=4,195)

**Studies excluded by title** (n=4,177)

**Selected studies**

(n=18)

**Studies excluded by abstract (n=5):**

Studies assessing environmental impact of complementary foods (n=2), and studies addressing environmental factors that facilitate breastfeeding (n=3)

**Full-text studies evaluated for eligibility**

(n=13)

**Studies excluded**

**(n=6)**

Studies assessing environmental impact of dairy products other than milk formula (n=1) and reviews or commentaries without quantitative data (n=5)

**Studies included from other sources**

(n=2)

**Included studies**

(n=7)

**Total sample**

**(n=9)**
